# DEVELOPMENTAL CHANGE IN PROBLEM DRINKING ACROSS THE LIFESPAN: BETWEEN- VERSUS WITHIN-PERSON AGE EFFECTS IN NATIONAL DATA

**DOI:** 10.1093/geroni/igad104.1887

**Published:** 2023-12-21

**Authors:** Matthew Lee, Ellen Yeung

**Affiliations:** Rutgers University New Brunswick, Piscataway, New Jersey, United States; George Washington University, Washington, District of Columbia, United States

## Abstract

When considering problem drinking from a lifespan developmental perspective, an often-stated underlying premise is that problem drinking peaks around early young adulthood and then declines throughout the remainder of the lifespan. However, relative to adolescence and young adulthood, the supposed later declines in midlife and older adulthood are less firmly established and based primarily on cross-sectional data. Thus, this study contrasted cross-sectional versus longitudinal age effects on problem-drinking changes across the adult lifespan. Analyses used two waves of longitudinal data from a U.S.-representative sample. We generated descriptive “porcupine figures” graphically depicting both cross-sectional and longitudinal age effects simultaneously. In addition, we used multilevel models to partition, test, and contrast cross-sectional versus longitudinal age effects on problem drinking in different age periods of the adult lifespan. While age effects were consistent in young adulthood across the cross-sectional and longitudinal tests, key discrepancies were shown in midlife and older adulthood. Specifically, the supposed continuation of problem-drinking reductions throughout midlife and older adulthood was observed cross-sectionally but not longitudinally. These findings call into question the notion of normative drinking-related declines that continue beyond young adulthood and throughout the remainder of the adult lifespan. We will discuss plausible alternative explanation for how cross-sectional data can lead to spurious conclusion about developmental change (e.g., age-confounded cohort effects). If replicated in future research, our evidence for relative stability or even increases in problem drinking in midlife and older adulthood can contribute to a more lifespan-developmentally-informed understanding of risky behaviors and thereby guide lifespan-developmental tailoring of interventions.

